# Supplementary material for: Mitochondrial AOX Supports Redox Balance of Photosynthetic Electron Transport, Primary Metabolite Balance, and Growth in Arabidopsis thaliana under High Light
Source: Int J Mol Sci. 2019 Jun 23;20(12):3067. doi: 10.3390/ijms20123067 (PMC6628045; doi:10.3390/ijms20123067)
Supplement: Supplementary file 1 [file ijms-20-03067-s001.pdf]

Supplementary Table S1. Primer sequences for the real-time PCR analysis

AGI No.	Gene name	Forward	Reverse
At1g07180	*NDA1*	5′-CTCCGTGAGAGCAAGGAAGG-3'	5′-GGCGAAGTGGAGGGGATATG-3'
At4g05020	*NDB2*	5′-ACTGACTCTCAAAGAGTTCC-3'	5′-CCGATTTGAACTCTTCGATC-3'
At3g54110	*UCP1*	5′-TCTGCTCTTGCTGGTGATGT-3′	5′-TACCCAGTGCACCTGTTGTC-3′
At1g22450	*COX6b*	5′-ATGGCGGATGCTGTGAACGC-3′	5′-CTCCGTCTTAACCTCTTCAG-3′
At3g53750	*ACT3*	5′-GGCTAACCGTGAGAAGATGA-3′	5′-CGACCTGCAAGATCAAGACG-3′

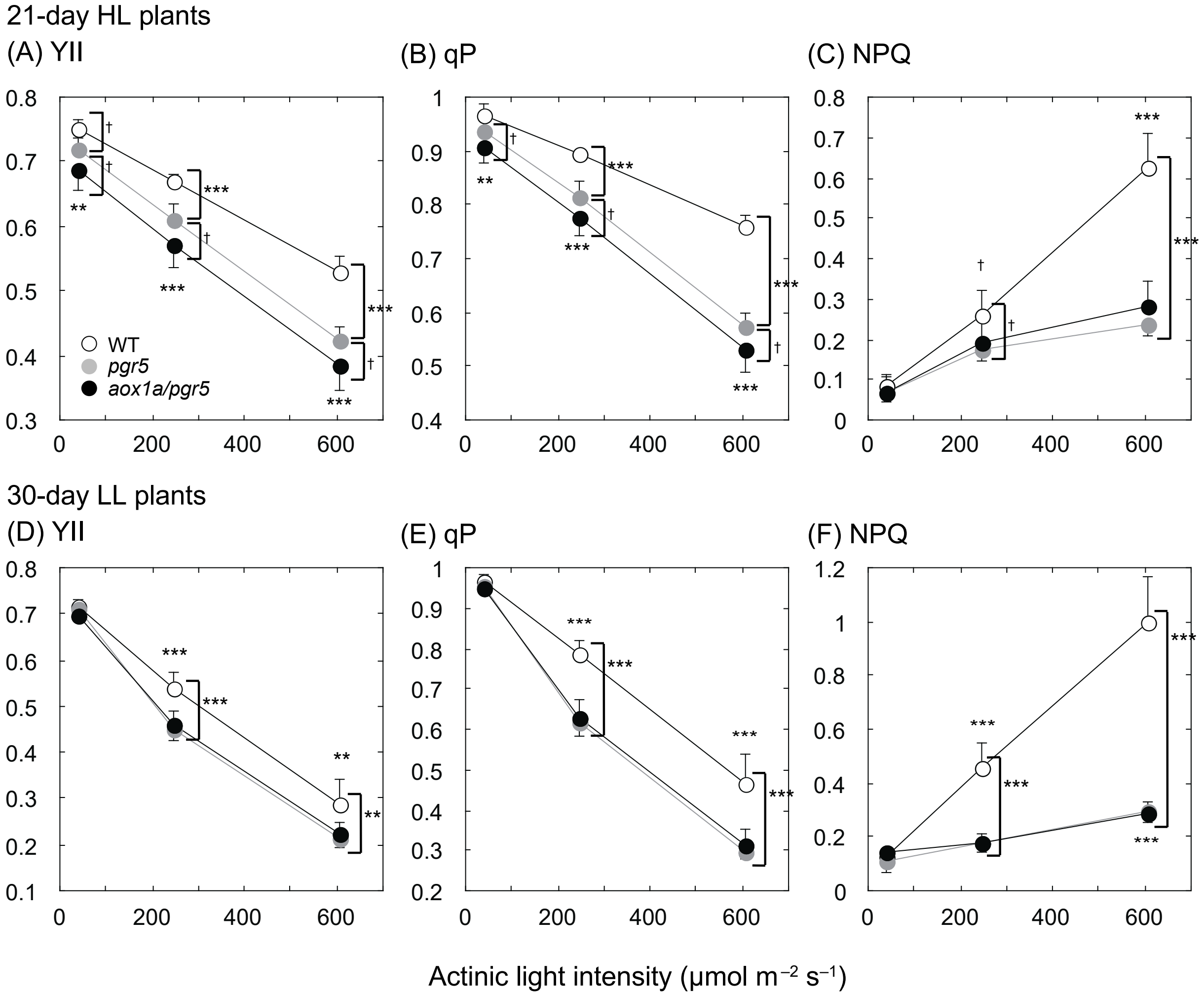

Supplementary Figure S1. Chlorophyll fluorescence parameters of photosystem II (PSII) of mature leaves of 21-day high-light (HL) and 30-day low-light (LL) plants. (**A,D**) Operating efficiency of PSII (YII); (**B,E**) photochemical quenching (qP); (**C,F**) non-photochemical quenching (NPQ). (**A–C**) HL plants; (**D–F**) LL plants. Means ± standard deviations are shown (n = 5). Results of one-way ANOVA are shown above or below three symbols, and results of Dunnett multiple comparison test are shown near parenthesis. † < 0.1, * < 0.05, ** < 0.01, *** < 0.001.

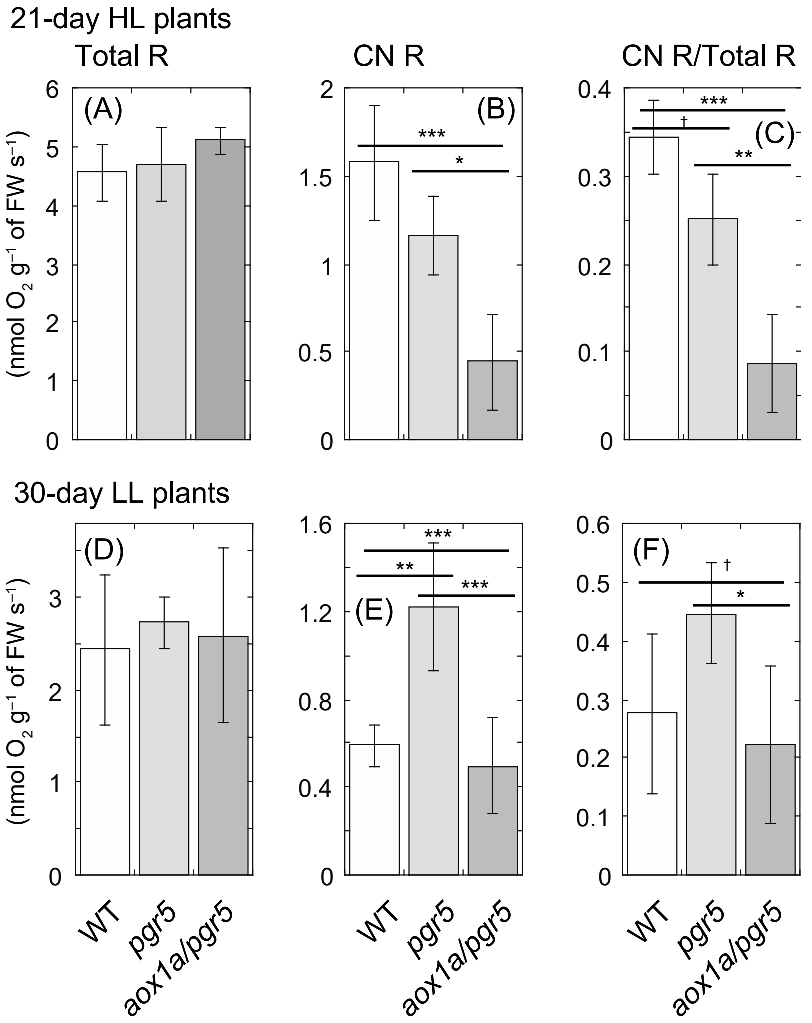

Supplementary Figure S2. Respiratory O_2 uptake rate of mature leaves of 21-day HL and 30-day LL plants. (**A,D**) O_2 uptake rate in the absence of inhibitor (Total R); (**B,E**) O_2 uptake rate in the presence of 2 mM KCN (CN R); (**C,F**) ratio of CN R to Total R (CN R/Total R). (**A-C**) HL plants; (**D-F**) LL plants. Means ± standard deviations are shown (n = 4-5). Results of one-way ANOVA are shown above three bars, and results of Dunnett multiple comparison test are shown above two bars. † < 0.1, * < 0.05, ** < 0.01, *** < 0.001.

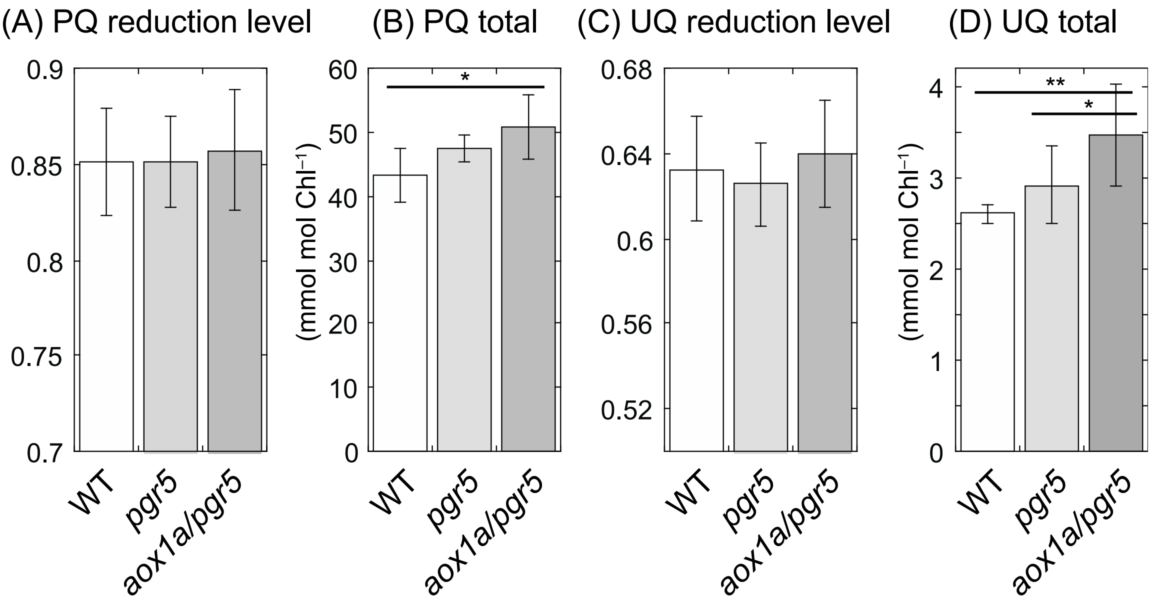

Supplementary Figure S3. Reduction levels of quinone and total amounts of quinones of mature leaves of 21-day HL plants. (**A**) PQ reduction level; (**B**) total amounts of reduced and oxidized PQ (PQ total); (**C**) UQ reduction level; (**D**) total amounts of reduced and oxidized UQ (UQ total). Means ± standard deviations are shown (n = 7). Results of one-way ANOVA are shown above three bars, and results of Dunnett multiple comparison test are shown above two bars. † < 0.1, * < 0.05, ** < 0.01, *** < 0.001.

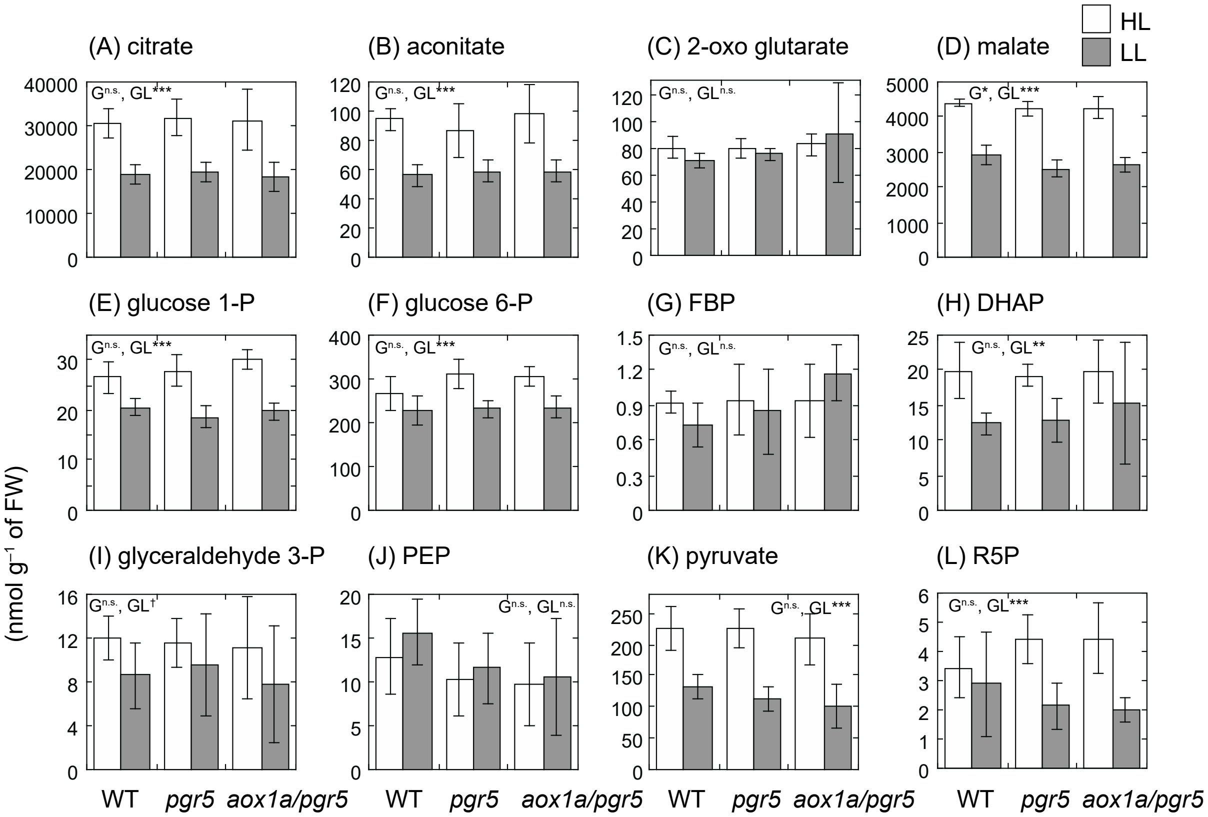

Supplementary Figure S4. Primary metabolite levels of shoots of 11-day HL and 15-day LL plants. Means ± standard deviations are shown (n = 5). Results of two-way ANOVA are shown in each panel. G and GL denote genotype and growth light factors, respectively, of two-way ANOVA. All probabilities of the interaction of two-way ANOVA are more than 0.05.. † < 0.1, * < 0.05, ** < 0.01, *** < 0.001.

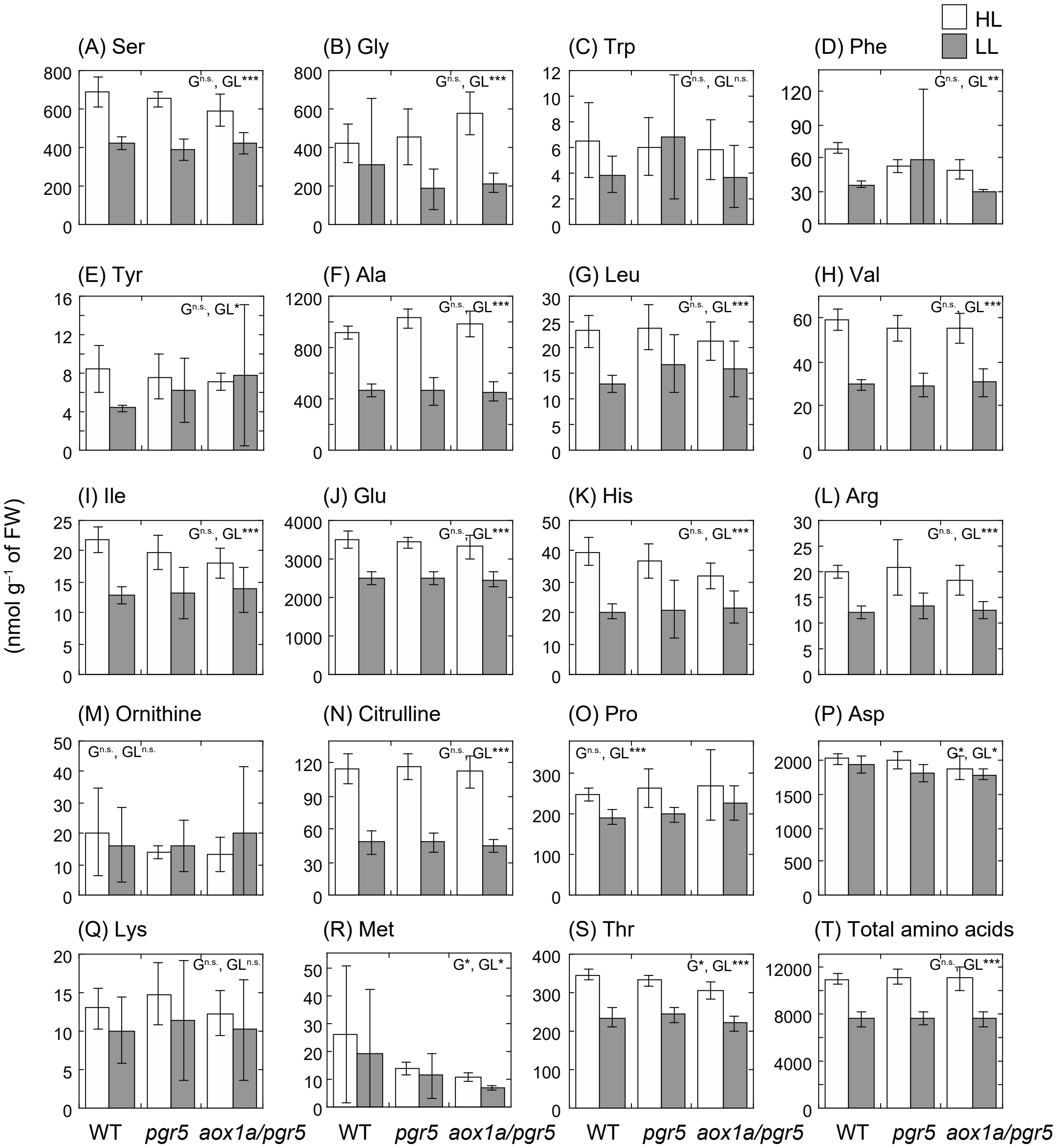

Supplementary Figure S5. Amino acid levels of shoots of 11-day HL and 15-day LL plants. Means ± standard deviations are shown (n = 5). Results of two-way ANOVA are shown in each panel. G and GL denote genotype and growth light factors, respectively, of two-way ANOVA. All probabilities of the interaction of two-way ANOVA are more than 0.05. † < 0.1, * < 0.05, ** < 0.01, *** < 0.001.
